# Retrospective study on safety and complications of direct percutaneous endoscopic gastrostomy in children below 10 kg

**DOI:** 10.1002/jpn3.70085

**Published:** 2025-05-22

**Authors:** Ilse J. Broekaert, Christoph Hünseler

**Affiliations:** ^1^ Department of Pediatrics, Faculty of Medicine and University Hospital Cologne University of Cologne Cologne Germany

**Keywords:** direct puncture PEG, infant, gastropexy, T‐fastener

## Abstract

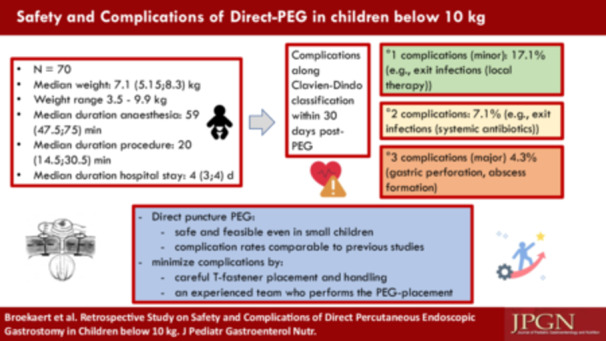

## 1 INTRODUCTION

Percutaneous endoscopic gastrostomy (PEG) insertion is often necessary in young infants and toddlers with swallowing disorders or increased calorie requirements to ensure enteral nutrition. The classic method is the pull‐through PEG (“pull‐PEG”), but alternative techniques such as the laparoscopic‐assisted PEG or the direct puncture technique (“push‐PEG”) may offer advantages including reduced stomal infection, better mobility, and easier changing. Previous studies^1^, ^2^, ^3^, ^4^ have shown that “push‐PEG” is not inferior to ‘pull‐PEG’. Here, we present our experience with PEG placement via the direct puncture technique with a focus on children with a body weight of less than 10 kg.

## 2 METHODS

All patients who underwent “push‐PEG” insertion between February 2016 and September 2024 at the University of Cologne Children's Hospital were identified from the hospital information system (via hospital coding system and patients' longitudinal medical record) for a retrospective cohort study. Only patients weighing less than 10 kg at the time of PEG insertion were included. We used an anchor set system with T‐fasteners to affix the stomach to the anterior abdominal wall facilitating primary placement of a PEG button with balloon. Demographic data (age, gender, diagnosis, and weight at PEG placement), procedural details (duration of the procedure, length of stay in hospital, and peri‐interventional antibiotics) and complications along the Clavien‐Dindo classification system (grade I: any deviation from the normal postoperative course, but without the need for any pharmacologic treatment; grade II: requirement of pharmacological intervention; grade III: need for surgical, endoscopic, or radiologic intervention) were analyzed retrospectively. Regarding the procedure, three T‐fasteners were applied regardless of the patient's weight and all patients received a 14 French PEG button with a stoma length adapted to the abdominal wall thickness (mostly 1.0–1.2 cm). The primary focus of this analysis was early postoperative complications within the first 30 days.

The descriptive statistics include mean values and standard deviations, medians and interquartile range (IQR) as well as percentages. The influence of age, weight and diagnostic group on complication rates was assessed using multivariate analysis. Statistical analysis was performed with SPSS version 29.0.1.

### 2.1 Ethics statement

Approval from the ethics committee of the University Hospital of Cologne was obtained.

## 3 RESULTS

Seventy children were identified at a median age of 346 days (IQR 184.25, 613.25). 47.2% (*n* = 33) were female, median weight was 7200 g (IQR: 5325, 8375), the minimum weight was 3500 g. The majority of infants were children with neuromuscular or complex genetic diseases (40%; *n* = 28) and patients with underlying renal disease (34.3%; *n* = 24). The median duration of PEG insertion was 20 min (IQR: 14.5, 30.5), 95.7% (*n* = 67) received peri‐interventional antibiotics (mostly ampicillin/sulbactam or a second or third generation cephalosporin) and the median hospital stay for elective inpatient PEG insertion was 4 days (IQR: 3, 4; *n* = 45). Table 1 provides an overview of the basic demographic data and the results.

**Table 1:** Basic information on patients and results.

*n* = 70	Mean (SD)	Median (IQR: 25, 75)
Basic information		
Age at PEG‐insertion (days)	310 (422)	346 (184, 613)
Weight (kg)	6.8 (1.98)	7.1 (5.15, 8.3)
Female % (*n*)	47.4% (33)	
Diagnosis % (*n*): Previous preterm infant neuromuscular/syndromic disease renal disease cardiac disease Cholestasis 1.4% (1)		8.6%, (6) 40.0%, (28) 34.3%, (24) 15.7% (11) 1.4% (1)
Results		
Duration anesthesia^a^ (min)	63 (24.5)	59 (47.5, 75)
Duration procedure (min)	23 (12)	20 (14.5, 30.5)
Duration hospital stay^b^ (days)	3.7 (1.4)	4 (3, 4)
Perioperative antibiotics % (*n*) Cefuroxime/ceftazidime Amoxicillin + clavulanic acid Piperacillin + tazobactam Ceftazidime No antibiotics		73% (51) 17% (12) 4.3% (3) 1.4% (1) 4.3% (3)
Complications (Clavien Dindo classification) during the first 30 days postprocedure % (*n*) Grade 0 Grade 1: Grade 2: Grade 3b:		70% (49) 17.1% (12) 7.1% (5) 5.7% (4)

Abbreviations: IQR, interquartile range; PEG, percutaneous endoscopic gastrostomy; SD, standard deviation.

a Only patients with PEG insertion as the sole procedure.

b Only for patients with planned admission for PEG insertion.

Early complications requiring surgical intervention occurred in four cases (5.7%) (gastric perforation *n* = 3, subcutaneous abscess *n* = 1). One child with postoperative hemorrhage at the PEG exit was treated with topical tranexamic acid. One child suffered from T‐fastener migration 3 weeks after the procedure, however, the T‐fastener was easily removed at the skin surface without complications. Tube dislocation did not occur. Grade 1 complications (17.1%; *n* = 12) consisted of superficial skin infections that could be treated locally with antiseptics. Grade 2 complications (7.1%; *n* = 5) included more extensive infections requiring systemic antibiotic therapy.

Multivariate analysis showed no correlation between age, weight or diagnostic group. The three patients who experienced gastric perforation had the following conditions: Costello syndrome (5500 g), trisomy 21 with atrioventricular septum defect (6000 g), and a genetic disease with chronic renal failure (9100 g).

## 4 DISCUSSION

Our data show that PEG placement via the direct puncture technique is feasible even in very small and young children. The acute complication rate within the first 30 days was 28.5%, with the majority being minor grade 1 and 2 complications, according to the Clavien Dindo classification. In four out of 70 cases (5.7%), there were serious grade 3b complications which required surgical intervention. In three patients (4.3%), persistent pneumoperitoneum was observed; in two cases (2.9%), gastric wall perforation by the T‐fasteners occurred; one patient (1.4%) experienced a leaky stoma with persistent air leakage. Despite careful precautions including controlled gastric air insufflation, minimal local anaesthetic to prevent tissue edema, and closure of retaining buttons at the end of the procedure, gastric wall perforation still occurred in three of these small patients. As this study is retrospective, minor complications such as skin irritation or mild pain may have been underreported, leading to a possible underestimation of the true complication rate. Previous reports^5^, ^6^ indicate that the thin metal rods of the T‐fasteners can perforate the stomach wall if excessive tension is applied. This was the case in two (2.9%) of our patients. To minimize this risk, T‐fasteners should be placed with caution, avoiding excessive tension, particularly in small children with thin stomach walls or compromised tissue perfusion, for example, in children with underlying cardiac disease. Since the direct puncture technique is more complex than the pull‐through technique, it should only be carried out by an experienced team. Our team typically consisted of two experienced paediatric gastroenterologists, an endoscopy nurse and, when available, a fellow in training. Sedation was administered by a paediatric anaesthesiologist and an anaesthesia nurse.

Overly tight T‐fasteners can also cause pressure‐related skin damage and local infections, complicating stoma care. In 17.1% (*n* = 12) of cases, the retaining buttons were actively removed 3–4 weeks after PEG‐placement, and some patients exhibited macerated or inflamed skin beneath the retaining buttons. However, no cases of early tube dislodgement were observed.

Comparing our results with previously published experiences of PEG placement via the direct puncture technique in children, our early complication rate of 28.5%, of which 5.7% were serious complications, aligns with previously reported rates for PEG placement vie the direct puncture technique in children. Kvello et al.^7^ reported a total of 45 gastrostomy related early postoperative complications in 87 patients (51.7%) of which the majority were grade I and II complications. Six patients (6.9%) experienced grade IIIb complications, including four (4.6%) requiring laparotomy and two (2.3%) requiring endoscopic procedures due to tube dislodgement, stoma infection, and leakage. Dahlseng et al. found a reduction in complications after implementing a standardized protocol, with only one (1.2%) serious acute complication (PEG dislocation) in 82 children.^8^ However, 44 patients (54%) experienced early gastrostomy‐related complications (54%), mostly peristomal infections, leakage and granulation tissue.^8^ The authors conclude that a detailed treatment protocol and limiting the number of physicians inserting the gastrostomy tubes reduced complication rates. Jean‐Bart et al. reported severe early complications in 5 of 679 children (0.8%) within the first week after PEG placement via the direct puncture technique including sepsis (*n* = 3; 0.4%), peritonitis (*n* = 2; 0.3%), and hemorrhage (*n* = 1; 0.1%) They identified younger age at the time of PEG placement as a risk factor for peristomal infection (odds ratio [OR]: 2.34 [1.03–5.30], *p* = 0.042). T‐fastener migration occurred in 17.3% of children, and neurological disease was protective (OR: 0.59 [0.37–0.92], *p* = 0.019).^9^ Scalise et al. observed a severe complication rate of 30% (*n* = 9) in 30 children, mainly due to tube dislodgement. Their cohort had a median age of 10 years and a median weight of 25 kg (10.7, 36.7).^10^ Göthberg and Björnsson reported two (1.0%) severe early complications in 206 children (mean age: 3.9 ± 4.7 years, mean weight 13.5 ± 9.9 kg), both involving tube dislodgement.^11^ Terry et al. performed PEG placement in 47 children (mean age: 6.4 years), with one (2.1%) major complication (gastrocolonic fistula) and one (2.1%) minor complication (early tube dislodgement).^12^

## 5 CONCLUSION

Our findings suggest that PEG placement via the direct puncture technique is safe and feasible in children weighing 3500–9900 g with complication rates comparable to previous studies. Careful T‐fastener placement and an experienced team who performs the PEG‐placement are essential to minimize risks. Further studies evaluating standardized protocols may help reduce complication rates and improve procedural outcomes.

## CONFLICT OF INTEREST STATEMENT

Ilse J. Broekaert received honoraria for presentations by Biogen and Pfizer. The remaining author declares no conflicts of interest.
